# Factors Leading to Diagnostic and Therapeutic Delay of Rheumatoid Arthritis and Their Impact on Disease Outcome

**DOI:** 10.7759/cureus.34481

**Published:** 2023-01-31

**Authors:** Umair Javaid, Tafazzul H Mahmud, Aflak Rasheed, Abd Ur Rahman Javaid, Saima Riaz, Amer Zohaib

**Affiliations:** 1 Rheumatology, Sheikh Zayed Postgraduate Medical Institute, Lahore, PAK; 2 General Medicine, University Hospital Monkland, Monkland, GBR

**Keywords:** health assessment questionnaire- disability index(haq-di), delayed referral, rheumatoid factor, rheumatoid arthritis, disease activity score (das-28)

## Abstract

Objective

To identify the factors which lead to delay in diagnosis and initiation of disease-modifying anti-rheumatic drugs (DMARDs) in rheumatoid arthritis (RA) patients and their impact on disease outcome and functional ability.

Methodology

This cross-sectional study was conducted from June 2021 to May 2022 at the Department of Rheumatology and Immunology, Sheikh Zayed Hospital, Lahore. Inclusion criteria were patients aged >18 years who were diagnosed with RA, based on American College of Rheumatology (ACR) criteria 2010. Delay was defined as any sort of delay which leads to delay in diagnosis or initiation of treatment of more than three months. The factors and impact on disease outcome were measured by using Disease Activity Score-28 (DAS-28) for disease activity and Health Assessment Questionnaire-Disability Index (HAQ-DI) for functional disability. The collected data were analyzed with Statistical Package for Social Sciences (SPSS) version 24 (IBM Corp., Armonk, NY, USA).

Results

One hundred and twenty patients were included in the study. Mean delay in referral to a rheumatologist was 36.75±61.07 weeks. Fifty-eight (48.3%) patients with RA were misdiagnosed before presentation to a rheumatologist. Sixty-six (55%) patients had the perception that RA is a non-treatable disease. Delay in diagnosis of RA from onset of symptoms (lag 3) and delay in start of DMARDs from onset of symptoms (lag 4) were significantly associated with increased DAS-28 and HAQ-DI scores (p-value 0.001).

Conclusion

The factors which led to diagnostic and therapeutic delay were delayed consultation with a rheumatologist, old age, low education status and low socioeconomic status. Rheumatoid factor (RF) and anti-cyclic citrullinated peptide (anti-CCP) antibodies had no role in diagnostic and therapeutic delay. Many RA patients were misdiagnosed with gouty arthritis and undifferentiated arthritis before consulting a rheumatologist. This diagnostic and therapeutic delay compromises RA management leading to high DAS-28 and HAQ-DI in RA patients.

## Introduction

Rheumatoid arthritis (RA) is a chronic, systemic inflammatory disease that adds significant morbidity and disability to the life of affected individuals [1]. An estimated 0.24-1% of the world's adult population is affected by RA, with a female-to-male ratio of 3:1 [2]. It mostly affects males in the sixth and seventh decade of their life while females of childbearing age [3]. The prevalence of RA is about 0.142% in the southern and 5.5% in the northern regions of Pakistan [4]. The point prevalence of RA has increased from 12.9% in 2011 to 26.9% in 2015 in Karachi [5]. Delay in diagnosis after onset of symptoms in initiation of treatment leads to quick disease progression and irreversible cartilage damage in less than two months. This leads to the formation of erosions on radiographs, followed by joint deformities in the next two years. Disease-modifying anti-rheumatic drugs (DMARDs) stand as the first line of treatment for rheumatoid arthritis as recommended by the American College of Rheumatology (ACR) [6]. It is reported that patients in earlier stages of the disease respond better than patients presented in delayed stages. Any delay in the initiation of therapy badly affects the disease outcome, progression, and functional disability of patients [7]. Initiation of treatment within 12 weeks of the start of symptoms was found to be the threshold for favorable and adverse disease outcomes [8]. Initiating treatment within 12 weeks of the onset of symptoms increases the chances of remission two-fold and decreases the use of biologic DMARDs from 32.24% to 10%, leading to better functional outcomes and declined disease progression rate [9,10]. It is a matter of great concern that only 8-42% of patients generally start treatment within 12 weeks of the onset of symptoms, as reported. Long-term studies have reported that 50% of patients with RA had to stop working within 10 years of the disease period which is 10 times the average rate caused by other diseases. The early stage of RA does not have classical clinical signs, responsible for a delay in diagnosis and initiation of therapy. A study by Hussain et al. showed an average time of nearly 30 months from the first consultation to the final diagnosis of RA [11]. A study by Santos-Moreno et al. showed that one-fifth of patients with a presumptive RA diagnosis was misdiagnosed by non-rheumatologists. A third of these patients were also receiving DMARD therapy, which leads to huge clinical and economic implications [12].

The RA patient treatment journey has four different stages: the onset of symptoms to consultation by a non-rheumatologist doctor (lag 1), the onset of symptoms to consultation by a rheumatologist doctor (lag 2), the onset of symptoms to RA diagnosis (lag 3), and the onset of symptoms to DMARD treatment initiation (lag 4).

The main aim of the study was to identify factors that lead to delay in diagnosis as well as treatment initiation in RA patients. This study was also designed to assess the impact of these delays on disease outcome and functional disability.

## Materials and methods

This cross-sectional study was conducted at the Department of Rheumatology and Immunology, Sheikh Zayed Hospital, Lahore, from June 2021 to March 2022, after getting approval from the institutional review board committee (approval szmc/irb/internal/00163/2022). World Health Organization (WHO) calculator was used to calculate the sample size which was 120 patients, with the level of significance set at 5%, the power of study was 80%. Disease Activity Score-28 Joints (DAS-28) in lag 3 and lag 4 were 4.04±1.37, and 3.9±1.4213 respectively. Inclusion criteria were patients aged >18 years, including both males and females diagnosed with RA according to ACR 2010 criteria [13], with raised erythrocyte sedimentation rate (ESR) and c-reactive protein (CRP) levels. Exclusion criteria were pregnancy, patients having any other disease which causes functional impairment, or patients having another connective tissue disease except RA. The non-probability consecutive sampling technique was used to select the patients for the study. For those patients who were fulfilling the eligibility criteria, informed consent was taken from them after explaining the whole procedure. Diagnostic and treatment delay has been defined as any sort of delay which leads to a delay in diagnosis or initiation of treatment of more than three months. This delay was calculated in four lag times. Lag 1: delay in a consultation with a non-rheumatologist doctor after the onset of symptoms, lag 2: delay in a consultation with a rheumatologist after the onset of symptoms, lag 3: delay in diagnosis of RA after the onset of symptoms, lag 4: delay in the start of DMARD therapy after onset of symptoms. Impact on disease outcome was measured by using DAS-28 for disease activity and Health Assessment Questionnaire-Disability Index (HAQ-DI) for functional disability. The results of DAS-28 were interpreted as remission <2.7, low disease activity 2.7-3.2, moderate disease activity 3.3-5.1, and high disease activity >5.1. HAQ-DI was interpreted as 0-1: mild to moderate disability, 1-2 as moderate to severe disability, and 2-3 as severe to very severe disability. According to delay in presentation to rheumatologist from onset of symptoms, patients were divided into three groups: group A included a delay of up to one year, group B included a delay of one to five years, and group C included a delay of more than five years.

The collected data were analyzed in the Statistical Package for Social Sciences (SPSS) version 24 (IBM Corp., Armonk, NY, USA). Descriptive statistics were calculated for qualitative and quantitative variables. Qualitative variables like gender and factors were measured as frequency and percentage. Quantitative variables like age were measured as mean and standard deviation. Lag times 1-4 were measured in the median and interquartile range. Mean DAS-28 and HAQ-DI were calculated for each group. Mann-Whitney U test and Kruskal Wallis H-test were applied to compare factors with median lag 3 and lag 4. in this study, a p value ≤0.05 was considered significant.

## Results

A total of 140 patients who met the inclusion and exclusion criteria were shortlisted. Twenty patients were later removed from the study, so 120 patients were included in the study. The mean age of patients was 38.99±11.53 years. There were 15 (12.5%) males and 105 (87.5%) females. Ninety-one (75.8%) patients were residents of urban areas. Forty-five patients (37.55%) had an education of secondary level. The mean visual analog scale (VAS) pain score at the first presentation was 8.43±1.13. One hundred and four (86.7%) patients were taking steroids before presenting to a rheumatologist. All demographics are mentioned in Table I.

**Table 1:** Demographics of patients Anti-CCP, anti-cyclic citrullinated peptide; CRP, c-reactive protein; ESR, erythrocyte sedimentation rate; IQR, interquartile range; RF, rheumatoid factor

Variables		Number(n) %
Number of patients		120
Age (years)		38.99±11.53
Gender	Males	15(12.5%)
	Females	105 (87.5%)
Resident	Rural	29 (24.2%)
	urban	91 (75.8%)
Education level	Primary	23 (19.2%)
	Secondary	45 (37.5%)
	Matric	37 (30.8%)
	Intermediate	8 (6.7%)
	Graduation	7 (5.8%)
Family type	Single	67 (55.8%)
	Joint	53 (44.2%)
Socioeconomic class	Poor	54 (45%)
	Middle	66 (55%)
Mean visual analog scale score for pain severity		8.43±1.13
Perception about rheumatoid arthritis	Treatable	54 (45%)
	Non-treatable	66 (55%)
Negative RF / Anti-CCP before presentation	Yes	38 (31.7%)
	No	82 (68.3%)
Negative ESR / CRP before presentation	Yes	7 (5.8%)
	No	113 (94.2%)
Steroids intake before presentation	Yes	104 (86.7%)
	No	16 (13.3%)
History of hakeem medication	Yes	82 (68.3%)
	No	38 (31.7%)
Misdiagnosis of rheumatoid arthritis	Yes	58 (48.3%)
	No	62 (51.7%)
Availability of Rheumatologist	Yes	84 (70%)
	No	36 (30%)
Mean delay in referral (weeks)		36.75±61.07
Delay in rheumatologist appointment (weeks)		3.42±0.87
Lag 1 median interquartile range(IQR) (months)		1(1.75)
Lag 2 median IQR(months)		12(114)
Lag 3 median IQR(months)		6(130)
Lag 4 median IQR(months)		13(60.88)

The mean delay in referral to a rheumatologist was 36.75±61.07 weeks. One hundred and thirteen (94.2%) patients had positive ESR and CRP status at presentation. Lag 2 when compared with lag 3 comes out to be significant (p-value 0.001). Sixty-six (55%) patients had the perception that RA is a non-treatable disease. Different factors causing a therapeutic and diagnostic delay in rheumatoid arthritis are mentioned in Table 2.

**Table 2:** Factors causing diagnostic and therapeutic delay in rheumatoid arthritis Anti-CCP, anti-cyclic citrullinated peptide; CRP, c-reactive protein; ESR, erythrocyte sedimentation rate; IQR, interquartile range; Lag 1, time from symptom onset to non-rheumatologist consultation; Lag 2, time from symptom onset to rheumatologist consultation; RF, rheumatoid factor

Variables		Lag 3 median (IQR) months	P value	Lag 4 median (IQR) months	P value
Age groups	<30 years	3(28)	0.001	4 (40)	0.001
	30-50 years	12(63)		18 (60)	
	>50 years	3 (1)		4 (2)	
Gender	Males	3 (9)	0.01	3.5(8.5)	0.005
	Females	6(27)		14(62)	
Resident	Rural	30 (78.5)	0.001	42(85)	0.04
	Urban	6 (9)		12(62)	
Education level	Primary	12 (11)	0.624	12 (10)	0.95
	Secondary	12 (79)		72 (72)	
	Metric	3(27)		42 (56)	
	Intermediate	3(27)		-	
	Graduation	3(27)		-	
Socioeconomic class	Poor	12 (27)	0.001	14(63)	0.01
	Middle	3(9)		12(56)	
Negative RA / Anti CCP before presentation	Yes	14(27)	0.88	42(46)	0.5
	No	6(9)		12(69)	
Negative ESR / CRP before presentation	Yes	12(27)	0.12	-	0.04
	No	9(15)		12(62.5)	
Availability of Rheumatologist	Yes	3 (9)	0.2	4 (8.5)	0.624
	No	12 (27)		18(62)	
Lag 1	0-6 months	6(11)	0.4	13(16.88)	0.14
	6-12 months	-		-	
	>12 months	-		-	
Lag 2	< 1 year	3(9)	0.001	4(8.75)	0.001
	1 -5 years	30(18)		42(24)	
	>5 years	6(82)		72(24)	

Rheumatoid factor (RF) and anti-cyclic citrullinated peptide (anti-CCP) antibodies were not significantly associated with any diagnostic or therapeutic delay. Negative ESR and CRP status were significantly associated with therapeutic delay but had no significance on diagnostic delay. Gender and place of residence were also significantly associated with diagnostic and therapeutic delay. Lag 3 was significantly associated with DAS-28 and HAQ-DI (p-value 0.001). Similarly, lag 4 had a significant association with DAS-28 and HAQ-DI (p-value 0.05 and 0.001 respectively). The association of DAS-28 and HAQ-DI with lag 3 and lag 4 is mentioned in Table 3.

**Table 3:** Association of lag 3 and lag 4 with DAS-28 and HAQ-DI DAS-28, Disease Activity Score-28 Joints; HAQ-DI, Health Assessment Questionnaire-Disability Index; Lag 3, time from symptom onset to rheumatoid arthritis diagnosis; Lag 4; time from symptom onset to disease modifying anti-rheumatic drug initiation

	Groups	Lag time	n	DAS-28	P-value	HAQ-DI	P-value
Lag 3	Group 1	<1 year	83	3.81 + 0.71	0.001	0.44 + 0.10	0.001
	Group 2	1 - < 5 years	16	4.5 + 0.51		0.65 + 0.05	
	Group 3	>5 years	21	5.0 + 0.83		0.90 + 0.08	
Lag 4	Group 1	<1 year	60	4.0 + 0.71	0.05	0.44 + 0.11	0.001
	Group 2	1-< 5 years	30	4.03 + 0.71		0.53 + 0.14	
	Group 3	>5 years	30	4.43 + 1.13		0.77 + 0.20	

## Discussion

In literature, it has been reported that early initiation of DMARDs in RA leads to a better outcome and less disease activity [14]. The diagnosis of RA and initiation of DMARDs therapy are generally started in settings where a rheumatologist is available. It is important to identify those factors which lead to delay in diagnosis from the onset of symptoms and initiation of treatment. 

In our study, it has been observed that low socioeconomic status is significantly associated with disease activity and poor disease outcome and is associated with therapeutic and diagnostic delay. A study by Verstappen et al. also reported that low socioeconomic status is associated with worse clinical and functional outcomes and low quality of life [15]. Camacho et al. also reported in their study that low socioeconomic status is associated with poor disease outcomes [16]. Cho et al. reported that patients having good socioeconomic status and higher education levels had early diagnoses and ultimately better disease outcomes in RA [17].

In our study, lag 2, i.e. delay in a consultation with the rheumatologist, was significant, showing that patients usually seek help from general physicians for treatment after the onset of symptoms and were not referred to a rheumatologist at the earliest step. A study by Irvine et al. also reported similar results, the main cause of delay was delayed referral to a rheumatologist by a general practitioner [18]. In our study, negative RF and anti-CCP antibodies had no significant relation with lag 3 and lag 4. A study by March et al. reported similar results that RF status has no significant association with diagnostic and therapeutic delay [19].

In our study lag 1 was one month (median) with an interquartile range (IQR) of 1.75 months which was shorter as compared to a study done in the USA and Saudi Arabia where it was 3.1 and six months respectively [11,20]. The median lag 2 in our study was 12 months, lag 3 was six months, and lag 4 was 13 months which was high when compared to the study done in the USA where lag 2 was 2.13 months, lag 3 was 2.91 months and lag 4 was 2.14 months [20]. Our lag times were less as compared to a study done in Pakistan where lag times reported were as follows: lag 1 was two months, lag 2 was 30 months, lag 3 was 12 months, and lag 4 was 18 months [13]. Our study showed that the higher the number of general physicians visited by the patient before presenting to a rheumatologist, the longer the delay in diagnosis of RA and start of DMARDs therapy (p-value 0.001). Hussain et al. also reported similar results that with an increasing number of doctors, there was a delay in lag 3 and lag 4 [11].

In our study, DAS-28 and HAQ-DI had a significant association with lag 3 and lag 4 (p-value 0.001). Badsha et al. reported in a study that DAS-28 was high in patients with diagnostic delay (p-value 0.02) [21]. Similarly, Kim et al. reported higher HAQ-DI scores in patients who had a delay in diagnosis when compared to patients in which diagnosis was made early (0.70±0.66 vs 0.6±0.63) [22]. A study by Ragab et al. reported that DAS-28 and HAQ-DI were low in patients in which therapy started within six months of the onset of symptoms [14]. In our study, RA was not properly diagnosed by general practitioners in 48% of patients and the majority of these patients were prescribed steroids improperly for symptomatic disease control.

The limitation of this study was that it was a single-center experience and we generally had patients of low socioeconomic status and lower education status as it was a public sector hospital. That's why factors responsible for the delay in high socioeconomic and high education status were not fully evaluated in this study.

## Conclusions

Major contributors to diagnostic and therapeutic delay in the management of RA were old age, low education, poor socio-economic status and late referral to a rheumatologist. About half of patients with RA who presented to a rheumatologist were previously misdiagnosed other than RA. This diagnostic and therapeutic delay leads to high DAS-28 and HAQ-DI in RA patients.
